# Case Report: A possible association between significant dissociations after esketamine treatment and histrionic personality disorder

**DOI:** 10.3389/fpsyt.2025.1666781

**Published:** 2025-12-17

**Authors:** Veronika Savica, Liene Sile, Maris Taube

**Affiliations:** 1 Faculty of Residency, Riga Stradiņš University, Riga, Latvia; 2 Department of Depression and Crisis, National Center of Mental Health, Riga, Latvia; 3 Department of Psychiatry, Riga Stradiņš University, Riga, Latvia; 4 Scientific Institute for Psychiatry, National Center of Mental Health, Riga, Latvia; 5 Department of Psychosomatic Medicine and Psychotherapy, Riga Stradiņš University, Riga, Latvia

**Keywords:** depression, dissociations, esketamine, histrionic personality, treatment

## Abstract

Dissociation is a complex and transdiagnostic phenomenon defined as a disruption of, or discontinuity in, the normal, subjective integration of one or more aspects of psychological functioning. Dissociation has been identified as one of the most frequently occurring adverse effects associated with the use of esketamine nasal sprays. Individuals with high trait dissociation may be at a higher risk of experiencing an induced dissociative state and a significantly higher risk of experiencing severe induced dissociation. High esketamine-induced dissociation may act as a predictor of optimal therapeutic response, rather than solely an adverse effect. This case report describes an unusually intense and persistent dissociative reaction in a patient with histrionic personality disorder (HPD) undergoing esketamine therapy. The patient’s marked dissociation upon treatment initiation raises the possibility that certain personality traits characteristic of HPD may heighten vulnerability to esketamine-induced dissociation. By explicitly examining a potential link between HPD and dissociation severity, this case highlights the importance of identifying patient-level factors that may amplify dissociative responses. Such knowledge has practical clinical implications for risk stratification, patient education regarding expected side effects, ensuring increased attention during the procedure.

## 1 Introduction

Dissociation is a complex and transdiagnostic phenomenon defined as a “disruption of and/or discontinuity in the normal, subjective integration of one or more aspects of psychological functioning, including – but not limited to – memory, identity, consciousness, perception, and motor control” (1).

In randomized controlled trials, dissociation was identified as one of the most frequently occurring adverse effects associated with the use of esketamine nasal sprays, and the severity of dissociation was found to be dose-dependent (2). While this adverse reaction is usually transient and resolves on its own without the need for medical intervention, it can cause discomfort for patients. Therefore, it is essential to educate patients about this potential side effect to ensure they are in a safe and calming environment that allows for the use of psychological interventions.

Although dissociation has often been characterized as an adverse effect of esketamine, growing evidence indicates that it may also serve as a predictive variable of therapeutic response. Several studies have demonstrated that greater dissociative intensity during ketamine or esketamine administration is associated with more robust short-term antidepressant effects, suggesting that dissociation may reflect engagement of neural mechanisms critical to treatment efficacy (3). While large-scale trials have reported mixed results, the overall pattern of findings supports the view that treatment-emergent dissociation may function as a potential biomarker of clinical response.

Recent multicenter and observational studies have shown that treatment-emergent behavioral and subjective responses to esketamine vary significantly across subgroups of patients. The paper by Martiadis V, et al. illustrates how individual traits and demographic factors can influence treatment-emergent emotional and behavioral responses, underscoring the broader principle that esketamine experiences are not uniform across patients (4). Similarly, prospective work focusing on psychological and cognitive dimensions, such as Olivola M, et al. suggests that individual differences in emotional–cognitive style may influence subjective states during treatment (5).

Identifying factors that may be associated with the severity of dissociation when using esketamine is clinically important as it would help predict the intensity of this side effect, allow clinicians to prepare and monitor patients, reduce patient distress, and may also provide insight into treatment response, since dissociation has been suggested as a potential predictive variable of therapeutic efficacy.The clinical case described here highlights a possible link between the severity of dissociation and histrionic personality disorder.

## 2 Case description

The patient was a 64-year-old female with a long-standing history of depressive episodes and a family history of depression. Her medical history includes diverticulitis, osteoporosis and obstructive sleep apnea. She is married and has a grown-up daughter. The patient has been unemployed for the past 3 years.

Early development progression was without any delay. The patient was raised in an intact family. She has older sister and younger brother. The mother was described as demanding and both emotionally and physically abusive, often neglecting patient’s needs and emotions. At the age of 16 patient attempted to cut her wrists after a conflict with her mother. She described this behavior as an attention seeking act, not a suicidal attempt. The patient excelled academically, consistently achieving top grades, and actively participated in various extracurricular activities. Peer relationships were established with ease. The patient has higher education in the field of culture and has held senior positions in public administration. Episodes of depression were initially triggered by job loss, deterioration of social status, and family relationship issues.

The patient experienced her first depressive episode in 2013, when her mother’s health started to deteriorate. Back then she was complaining of anxiety, lack of energy, apathy, difficulties concentrating, low mood as well as feelings of derealization. During her first hospitalization there was an improvement in symptoms, she became more active, her mood improved. But it was also noted that she was controversial and rather dramatic, each day describing different symptoms, one day complaining of extreme anxiety and the day after denying these symptoms. After her first discharge she was referred to outpatient clinic to continue pharmacological treatment. She started experiencing recurrent depressive episodes since then; she has been hospitalized seven times for depression in total. Each depressive episode was characterized by a severely low mood, psychomotor retardation, anhedonia, poor sleep and appetite, extremely low self-esteem, feelings of being useless and a burden to others, as well as suicidal thoughts and plans. She tried to overdose medication, but the attempt was interrupted by her sister. The patient had undergone a range of different psychopharmacological and psychological treatments throughout her life, that were determined by Latvian clinical algorithms (6).

During past depressive episodes, the patient received comprehensive treatment during inpatient stays and outpatient care. The patient has undergone an extensive range of pharmacological treatments, receiving the full course and maximum tolerated doses, including escitalopram, fluoxetine, duloxetine, venlafaxine, clomipramine, bupropion, vortioxetine, paroxetine, agomelatine, mirtazapine, and trazodone, as well as various combination regimens with aripiprazole and cariprazine, before treatment was advanced to esketamine. The patient also received psychotherapeutic treatment (cognitive behavioral therapy), and art therapy (visual art, music therapy, dance movement therapy, drama therapy). The patient often complained about various side effects from medication, for example, Trazodone has provoked allergic rhinitis, other medication made patient unstable, and she was losing her balance. She also reported a feeling that no medications have had any positive long-standing effects. Depressive symptoms had waxed and waned with different treatments but had never reached full remission and in the last years had progressively worsened, resulting in repeated hospital admissions. Physical and neurological examinations, laboratory testing, cranial magnetic resonance imaging as well as computer tomography had been performed since disorder onset but had never shown any significant abnormality that could be causally linked to depressive symptoms.

At intake, patient appeared to be hipomimc, her speech was low and quiet. She complained of low mood, anhedonia, social isolation and decreased appetite. Low mood was persistent throughout the day with associated lethargy and lack of interest. Before admission she was thinking of committing suicide by overdosing medication. She stated that she experienced the feeling of excessive, self-centered sorrow because she could not attend the event she was invited to. She started crying and decided to take an increased dosage of medication but her suicidal attempt was interrupted by the call of relative who supported her emotionally and advised to take care of herself. During the psychiatric assessment interview she mentioned several times that it was a ‘pure miracle’ that her relative called at the right time. During the interview traits of theatricality were observed. There was no evidence of hallucinations or delusional ideas.

Considering lack of clinical effect and intolerance of multiple previous pharmacological combinations, and depression progressively worsening, we evaluated further therapeutic options.

Since November 2024, esketamine has been fully reimbursed by the state in Latvia, and the patient began esketamine therapy in January 2025 in a psychiatric inpatient unit, considering that our previous diagnostic workup (neurological examinations, electrocardiography, standard laboratory blood tests) had not shown any contraindications. She remained hospitalized for 22 days, during which she received eight intranasal esketamine inhalations (twice weekly during the first month). Subsequently, the patient continued receiving esketamine on an outpatient basis, once weekly for one month, and then once every two weeks. The frequency and dosage of esketamine administration received by the patient, as well as her Clinical Global Impressions-Severity of Illness, Clinical Global Impressions-Improvement, and Montgomery-Åsberg Depression Rating Scale (MADRS) scores are presented in **Table 1**.

**Table 1:** Dates, dosages, scales (MADRS, CGI-S, CGI-I) and dissociation severity during the esketamine treatment course.

Date	Dose (esketamine)	MADRS	CGI-S	CGI-I	Severity of dissociations
15/01/2025	56 mg	36	6	3	Very severe
17/01/2025	56 mg		5	4	Very severe
21/01/2025	84 mg		5	4	Very severe
24/01/2025	84 mg		5	3	Less severe
28/01/2025	84 mg		4	4	Less severe
31/01/2025	84 mg		4	3	Less severe
4/02/2025	84 mg		3	4	Less severe
7/02/2025	84 mg		3	4	Less severe
13/02/2025	84 mg		3	4	Less severe
20/02/2025	84 mg		3	4	Less severe
27/02/2025	84 mg		3	4	Less severe
6/03/2025	84 mg	16	3	4	Less severe
21/03/2025	84 mg		3	4	Less severe
2/04/2025	84 mg		3	4	Less severe
6/04/2025	84 mg		3	4	Less severe
30/04/2025	84 mg		3	4	Less severe
14/05/2025	84 mg		3	4	Less severe
2/06/2025	84 mg		3	4	Less severe
18/06/2025	84 mg		3	4	Less severe

CGI-I, Clinical Global Impressions-Improvement; CGI-S, Severity of Illness; MADRS; Montgomery-Åsberg Depression Rating Scale.

Therapy was initiated with 56 mg of esketamine. After the first inhalation, the patient reported pronounced dissociative symptoms. She reported seeing a brightly colored bird flying into the room, seeing through the wall into the neighboring patient’s room, and perceiving herself from an outside perspective. She reported feeling as though she had a third eye on her forehead, that her chest was as large as ‘mountains,’ her teeth were enormous, and her arms were small and thin. The symptoms persisted for approximately 80–90 minutes.

Despite these experiences, the therapy had a positive effect, leading to a decision to increase the dose to enhance a potentially better therapeutic outcome. The dose was increased to 84 mg of esketamine. Upon increasing the dose, the dissociative symptoms became even more intense- she felt as if there was a pond with ducks inside her stomach. The duration of symptoms was almost the same as before, lasting for approximately 80–90 minutes. While continuing treatment with 84 mg esketamine, the dissociative experiences persisted, although they were slightly less intense.

Following discharge from inpatient care, the patient continued esketamine therapy on an outpatient basis—once a week for one month, and then once every two weeks—in accordance with the treatment protocol. Before each treatment session, the patient reported experiencing very mild anticipatory anxiety, which progressively diminished over subsequent administrations. Initially, the dissociative symptoms elicited anxiety and subjective discomfort; however, as the patient received reassurance and more detailed psychoeducation regarding the nature of these symptoms, her sense of safety increased, and the dissociation no longer interfered with functional recovery. Each dissociative episode persisted for approximately 80–90 minutes, with minimal variability in duration across sessions. Dissociative symptoms occurred after every inhalation and did not decrease in intensity.

## 3 Diagnostic assessment

The patient’s diagnosis of depression was based on the symptoms defined in the International Classification of Diseases 10th edition (ICD-10) (7) (i.e., low mood, lack of interest, psychomotor retardation, low self-esteem, sleep and appetite disturbances, and suicidal thoughts). Her depressive episodes lasted at least two weeks and were recurrent, which supported the diagnosis of recurrent depressive disorder.

The patient also exhibited traits characteristic of a histrionic personality disorder. She met the criteria for a specific personality disorder, characterized by marked deviations in cognition, affectivity, and interpersonal functioning. These behavioral deviations were evident across various social contexts, including home, relationships with relatives, and within the treatment setting. The deviations were associated with significant personal distress and are believed to have originated in early childhood, likely as a result of lack of attention and positive reinforcement from her mother. The observed symptoms cannot be attributed to the organic brain disorder or dysfunction. She displayed unstable affective responses, a tendency to overdramatize events, a strong desire to be the center of attention, and a need to feel special. She dressed flamboyantly, with extensive use of accessories, exhibited self-focused speech, self-praise, and symptom dramatization. She previously worked as a cultural project manager and was consistently in the public spotlight. During treatment, she was unemployed and subsequently felt a lack of social attention. Based on ICD-10 criteria, these traits support a diagnosis of histrionic personality disorder (four out of six diagnostic criteria according to the ICD-10 were met- Self-dramatization, theatricality, or exaggerated expression of emotions; Suggestibility, easily influenced by others or by circumstances; Continually seeks excitement and activities in which the subject is the centre of attention; Inappropriately seductive in appearance or behaviour; Overly concerned with physical attractiveness.) (8). Additionally, the psychological assessment was conducted by psychologist in February 2025. The assessment included the use of methods such as a semi-structured interview, 10-word memorization, pictograms, Schulte tables, the odd-one-out task, the Montreal Cognitive Assessment (MoCA), and the Minnesota Multiphasic Personality Inventory – Second Edition (MMPI-2).

According to the MMPI-2 validity scale results, the test was not interpretable due to the patient’s tendency to exaggerate symptoms. Personality assessment indicates a tendency to exaggerate difficulties and to respond to situations in an overly intense or exaggerated manner. The MMPI-2 profile was deemed non-interpretable due to compromised validity indices, precluding any formal psychological interpretation. Nevertheless, we reference select descriptive features of the profile to provide contextual information about the patient’s presentation. These observations should be understood as tentative and limited, given the invalid overall profile.

Given that dissociation severity is central to the present hypothesis, it is important to note that we were unable to assess dissociation using standardized measures such as the CADSS. A validated Latvian version of the CADSS is currently unavailable, and therefore dissociation was evaluated through clinical observation rather than structured assessment. This methodological limitation should be considered when interpreting the findings (9).

To initiate esketamine therapy in Latvia, specific eligibility criteria are applied to optimize limited healthcare funding. Therapy can be initiated in patients with treatment-resistant depression, in combination with SSRIs or SNRIs. A MADRS score of at least 28 is required before therapy can be initiated, and the decision to begin treatment is made by a board of three psychiatrists. Additionally, after eight weeks of esketamine therapy, the MADRS score must decrease by at least 50% for the treatment to continue for an additional four months.

In this case, the patient met the criteria for initiating esketamine therapy. Her MADRS score prior to therapy was 36, and after eight weeks of treatment, it had decreased to 16, which permitted continuation of the therapy. She concurrently received 20 mg of the SSRI antidepressant, fluoxetine.

## 4 Discussion

Dissociation is observed across various psychiatric disorders. A recent meta-analysis revealed that dissociative symptoms are most prevalent in dissociative disorders (e.g., dissociative identity disorder, dissociative amnesia, depersonalization/derealization disorder, dissociative fugue, and dissociative disorder not otherwise specified), followed by post-traumatic stress disorder, borderline personality disorder, and conversion disorder (10). Real-world research—such as the Italian multicenter REAL-ESKperience study has documented considerable variability in dissociative intensity and subjective experiences depending on patient characteristics and psychological profiles (11). Severe and chronic stress, particularly in the form of childhood abuse and neglect, has been strongly implicated in the emergence of dissociation. These findings point to a complex interaction between genetic, neurobiological, and cognitive vulnerabilities and exposure to stressful life events (12, 13). Although the exact neural mechanisms underlying dissociation remain incompletely understood, recent advances in neuroimaging have revealed consistent alterations in brain regions associated with emotion processing and memory, attention and interoceptive awareness, filtering of sensory input, self-referential process, cognitive control and arousal modulation (14).

It is possible that dissociation should be understood not solely as a tolerable side effect, but as a process that may itself contribute therapeutically, especially in certain diagnostic populations. High levels of dissociation due to the use of intravenous ketamine and esketamine may serve as a predictor for the most effective therapeutic response. Therefore, identifying individuals who are more likely to experience pronounced dissociative symptoms may be clinically useful. Some studies have suggested that dissociation induced by these drugs is linked to a better antidepressant response (15–17), while others have not found this association (18–20). In one study that measured levels of trait dissociation, individuals with high trait dissociation were found to have a higher risk of experiencing an induced dissociative state and a significantly higher risk of experiencing severe induced dissociation. These results suggest that trait dissociation may serve as a predictor for induced dissociation in patients with treatment-resistant depression when using ketamine or esketamine (21). The case series by Sarasso P, et al., describes dissociative and disembodiment-like experiences in a subgroup of depressed patients with depersonalization features, demonstrating patient’s vivid perceptual distortions and altered body schema (22).

According to the Diagnostic and Statistical Manual of Mental Disorders, 4th edition (23), individuals with histrionic personality disorder are highly suggestible, meaning they are easily influenced by others or external circumstances. Hence, individuals with this personality disorder may be at a high risk for experiencing dissociative symptoms when using intranasal esketamine, as their trait dissociation levels may be initially high (24). However, data on the prevalence of dissociation in different personality disorders remains limited.

### 4.1 Limitations

Histrionic personality traits may represent only one of several factors contributing to the patient’s pronounced dissociative experiences following esketamine administration, and no causal inference can be drawn from a single case. Environmental and contextual variables—such as the inpatient treatment setting, the patient’s expectations regarding esketamine, and her level of emotional arousal prior to dosing—may also have modulated the intensity of the dissociative response and should be considered when interpreting the findings. Moreover, dissociation was not assessed using standardized quantitative instruments such as the Clinician-Administered Dissociative States Scale (CADSS), as no validated Latvian version is currently available.

## 5 Conclusion

This hypothesis-generating case report suggests a possible association between histrionic personality traits and heightened esketamine-induced dissociation. As no causal conclusions can be drawn from a single case, systematic studies in larger cohorts are required to evaluate this preliminary observation. Improved understanding of a patient’s personality traits may help psychiatrists predict the degree of dissociation, provide timely information to the patient, and ensure close monitoring during esketamine administration, thus reducing patient distress and predicting best therapeutic outcome.

## 6 Patient perspective

The patient has continued esketamine therapy with sustained improvement. She noted that previous to treatment, she lived as if in a ‘cage,’ avoiding social contact, but now goes outside and communicates with others. Suicidal thoughts and feelings of life being meaningless have disappeared. While dissociative symptoms continue to persist after every esketamine dose, the patient is now prepared for them and experiences less distress than when therapy was first initiated.

## Data Availability

The original contributions presented in the study are included in the article/supplementary material. Further inquiries can be directed to the corresponding author.
